# System alignment supports cross-domain learning and zero-shot generalisation

**DOI:** 10.1016/j.cognition.2022.105200

**Published:** 2022-10

**Authors:** Kaarina Aho, Brett D. Roads, Bradley C. Love

**Affiliations:** a University College London, Department of Experimental Psychology, 26 Bedford Way, London WC1H 0AP, United Kingdom; b The Alan Turing Institute, 96 Euston Road, London NW12DB, United Kingdom

**Keywords:** Learning, Mapping, Relational similarity, Alignment, Computational modelling

## Abstract

Recent findings suggest conceptual relationships hold across modalities. For instance, if two concepts occur in similar linguistic contexts, they also likely occur in similar visual contexts. These similarity structures may provide a valuable signal for alignment when learning to map between domains, such as when learning the names of objects. To assess this possibility, we conducted a paired-associate learning experiment in which participants mapped objects that varied on two visual features to locations that varied along two spatial dimensions. We manipulated whether the featural and spatial systems were *aligned* or *misaligned*. Although system alignment was not required to complete this supervised learning task, we found that participants learned more efficiently when systems aligned and that aligned systems facilitated zero-shot generalisation. We fit a variety of models to individuals' responses and found that models which included an offline unsupervised alignment mechanism best accounted for human performance. Our results provide empirical evidence that people align entire representation systems to accelerate learning, even when learning seemingly arbitrary associations between two domains.

## 1 Introduction

Learning is often viewed as event-based. For example, pairing a face with a label provides a means to learn a stranger's name. A complementary possibility is that humans learn by establishing correspondences between entire *systems* (Goldstone & Rogosky, 2002).

Imagine you are abroad with your partner who is watching a basketball game on television in an unknown language. You are facing away from the television unpacking your luggage. You frequently hear cheering followed by the announcer saying various utterances containing “Michael”. Your partner, noticing your disinterest in the game, plugs their headphones into the television. Turning toward the muted television, you notice the same star player from the home team keeps scoring. Despite being limited to asynchronous cross-modal input, a reasonable inference based on aligning systems is that the star player's name is Michael.

Mappings like this are possible far beyond simple features like frequency. For instance, similarity relations across visual and linguistic systems may mirror one another: cups and mugs appear in related linguistic contexts concerning drinking and also are visually similar. The semantic similarity of cups and mugs is a latent factor here, causing them to be both (a) discussed in similar ways and (b) similar in appearance. Any pair of systems possessing common structure like this could enable the use of similarity relationships to perform accurate cross-system mappings.

**Fig. 1:**
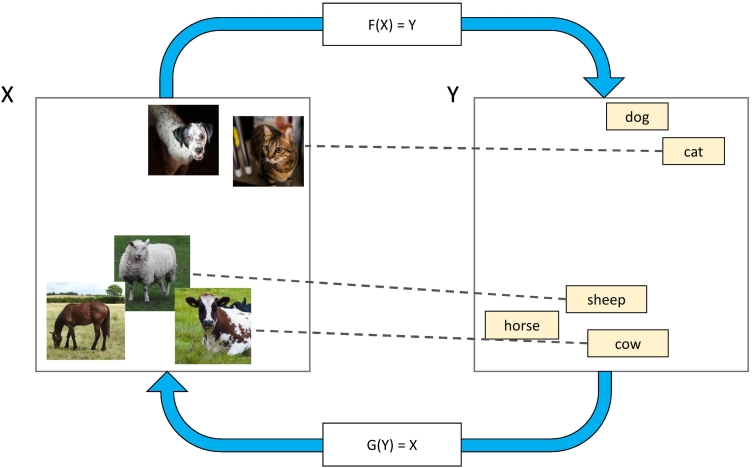
Visualisation of system alignment. Notice that the similarity relationships in the visual and linguistic domhains mirror one another. This shared structure is a requirement for systems to be alignable. Functions *F* and *G* learn correspondences between entire domains *X* and *Y*. Dashed lines represent known mappings for individual items. In this example, no mapping is known for “horse” or “dog”, but the correct mapping for these items could be inferred in an unsupervised fashion based on the alignment of systems via *F* and *G*. This demonstrates how system alignment may facilitate generalisation.

It has been shown that information exists in the environment to support aligning conceptual systems based on similarity relations. Roads and Love (2020) conducted an information analysis on different unimodal embeddings, which found that similarity relations remain consistent across modalities. That is, if “cat” and “dog” occur in similar linguistic contexts, their corresponding referents are likely to occur in similar visual contexts. We henceforth refer to the use of similarity relations to perform cross-system mappings as *system alignment* (Fig. 1).

We define a *system* as a set of items organised within a *domain*, where a domain is the set of possible inputs to a mapping function *F*(*X*) for a given task (see Fig. 1). In learning to label visual objects, we learn correspondences between systems of representations in visual and linguistic modalities. In this case, the modalities are the relevant domains.^1^ Although our study will focus on perceptual and spatial domains, we intend our contribution to be general and predict that it will apply to other cases in which humans could capitalize on cross-system structure to facilitate learning.

Research in analogy seeks alignments between representations (Doumas, Puebla, Martin, & Hummel, 2020; Gentner, 1983; Holyoak & Thagard, 1989; Lu, Chen, & Holyoak, 2012), but whereas analogical alignment is between two analogs, such as an atom and the solar system, we suggest that entire conceptual systems could be aligned. System alignment also diverges from alignment work in category learning (Lassaline & Murphy, 1998) and in similarity perception (Goldstone & Medin, 1994), as it does not require features to be shared across systems for mapping, and depends instead on the similarity relationships within systems.

System alignment offers a possible explanation for humans' remarkable success in acquiring multimodal concepts, despite this being a famously challenging and underconstrained task. Infants can acquire an understanding of more than 300 concepts by 16 months of age (Fenson et al., 1994). Yet even the most *supervised* learning episodes—such as pointing at an object while naming it aloud—are ambiguous. This problem of referential ambiguity is demonstrated by Quine's famous thought experiment (Quine, 1960); if a teacher points at a rabbit hopping through a field and says “gavagai” aloud to a naive learner, how does the learner know what “gavagai” refers to? It could mean hopping, rabbit, fur, field - the list of possibilities goes on.

A number of constraints on direct word learning are known, including the whole-object assumption, the mutual exclusivity assumption and the taxonomic assumption (Golinkoff, Hirsh-Pasek, Bailey, & Wenger, 1992; Markman, 1994; Markman & Hutchinson, 1984; Merriman, Bowman, & MacWhinney, 1989). System alignment could offer an additional constraint, and could even facilitate cross-modal learning *offline* (that is, in the absence of synchronous input across systems) by capitalising on common structural relationships. For example, the systems in Fig. 1 could be mapped by matching the similarity relationships between concepts across domains, requiring no synchronous input across modalities. As such, system alignment can explain learning from ambiguously supervised events (such as those discussed in the “gavagai” problem), and even in the absence of explicit instruction (Cartmill et al., 2013; Lieven, 1994; Samuelson, Smith, Perry, & Spencer, 2011). While many informative learning episodes will be *online* (i.e., synchronous input across systems, such as in direct instruction where items mapped across systems are presented together), system alignment opens up an additional raft of offline learning opportunities.

**Fig. 2:**
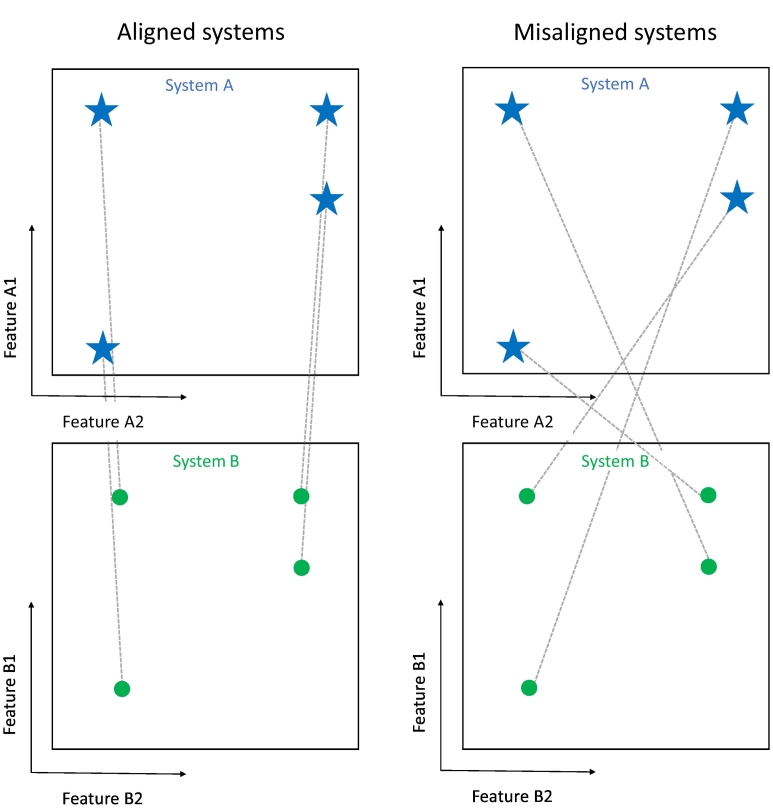
Examples of aligned and misaligned systems. In aligned systems, similarity relations are recapitulated across systems, which is not true for misaligned systems.

While system alignment enables purely unsupervised learning, signals about the strength of alignment may also enhance learning in the presence of supervised examples, as memory of individual item mappings is reinforced by the alignment of systems. In this study, we aimed to investigate whether participants were better able to learn associations between aligned systems compared to misaligned ones in a supervised learning task (Fig. 2). Aligned systems are those for which the correct pairing of objects between systems is dictated by their second-order isomorphism. This means paired items share a pattern of relationships within their respective systems, while sharing no physical properties (Shepard & Chipman, 1970). In a misaligned set of systems, paired items share neither physical properties nor patterns of relationships.

Our primary hypothesis is that learning will be facilitated when systems align, even in cases where feedback is provided and synchronous. That is, even when system alignment is optional for success in the learning task, people will engage in it. In the current experiment, this yields the prediction that participants will show improved learning for cross-system associations when systems are aligned than when they are misaligned. A default system alignment strategy might produce idiosyncratic error patterns for misaligned scenarios.

System alignment should create expectations for how an unseen example maps from domain *X* to *Y* based on its relationships to other items in *X* (Fig. 1). This form of generalisation can be described as *zero-shot generalisation* (Xian, Schiele, & Akata, 2017), because the items in domain *X* and *Y* are both novel and their pairing can only be inferred through their relationships to experienced items within their system. Notice this form of generalisation differs from forms of generalisation commonly considered, such as in category learning where the item is novel but the category label is not. We predict that participants who align systems should be able to perform zero-shot generalisation to a novel stimulus in *X* to *Y*, which would be like knowing the name of visual object one has never encountered before. Finally, we predict that a computational model including an offline alignment mechanism would be the best fit for participants in the aligned condition, compared to models simulating (i) rote-memorisation and (ii) cross-system mapping with no distributional alignment.

## 2 Experiment

We tested our hypotheses using a paired-associate learning (PAL) paradigm, presented as a memory game. We conducted an online experiment via Amazon Mechanical Turk (AMT), in which participants learned to associate cartoon monsters with their homes on a map.

Monsters were presented one at a time, and the underlying structure of the correct answers assigned to a given participant was either aligned or misaligned (cf. Tompary & Thompson-Schill, 2021). For generality, we varied the rotation of the neighbourhood map between subjects. Details of the rotation condition are included in Appendix B.

### 2.1 Methods

#### 2.1.1 Participants

AMT participants (*N* = 493) limited to the US and Canada completed the experiment. We required participants to have completed ≥ 1000 prior tasks with an acceptance rate ≥ 95%. All participants provided their informed consent prior to participation. The task took approximately 15 minutes to complete and participants were paid $2.00 for their participation.

One participant was excluded from analysis for submitting inaccurate demographic responses. 49 further participants were excluded for poor engagement (details in Appendix D). This resulted in *N* = 443 participants whose responses were analysed. The sample was 39.5% female and age ranged from 20 to 72 years (*M* = 38*.*5, *SD* = 10*.*7).

#### 2.1.2 Materials and design

The study had a 2 × 2 (system alignment × rotation) design, where alignment and rotation were both between-subject factors. Each participant completed 5 blocks of trials. One further trial tested generalisation to an unseen stimulus. Participant assignments across the four experimental conditions were counterbalanced.

House stimuli varied in their *x* and *y* positions on the neighbourhood map. Monster stimuli varied on two dimensions: body colour and eye orientation, where their eye was an orientation grating. Eye orientation took values between 5° and 85° from the horizontal, and body colour took values along a perceptually uniform trajectory from blue to green.^2^ Details of stimulus features are provided in Appendix B.

**Fig. 3:**
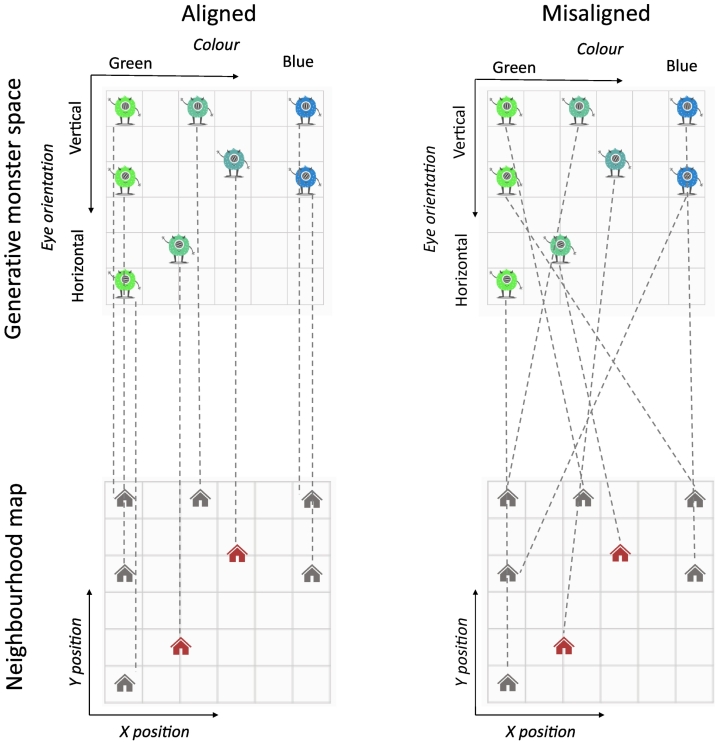
Examples of the correct mappings in each alignment condition. Monsters vary in colour and eye orientation; houses vary in their *x* and *y* coordinates on the neighbourhood map. In the aligned condition (left), the relationships between monsters' features (e.g. two steps more blue) were mirrored in the spatial relationships between their assigned homes (e.g. two steps horizontally). The red houses and the corresponding monsters were only shown at the end of learning to evaluate zero-shot generalisation based on system alignment. Grid lines are shown for reference only, and were not visible on the neighbourhood maps during the experiment. Note that participants never saw all monsters in their correct homes: monsters were only ever shown in their correct homes one at a time. (For interpretation of the references to colour in this figure legend, the reader is referred to the web version of this article.)

Monster stimuli were selected from positions in the 2D feature space which corresponded with the six house positions on the neighbourhood map. For participants in the misaligned condition, the stimuli in this constructed set were randomly assigned to houses in the neighbourhood (see Fig. 3).

#### 2.1.3 Procedure

##### 2.1.3.1 Pre-exposure

After being briefed, participants were shown two animations which cycled through the full range of feature values for monster colour and eye orientation respectively. Six feature values were shown for 1000ms each on a loop. This aimed to familiarise participants with the monster stimulus space. Accordingly, the instructions on the page drew participant attention to the two dimensions of monster stimulus variation.

##### 2.1.3.2 PAL task

**Fig. 4:**
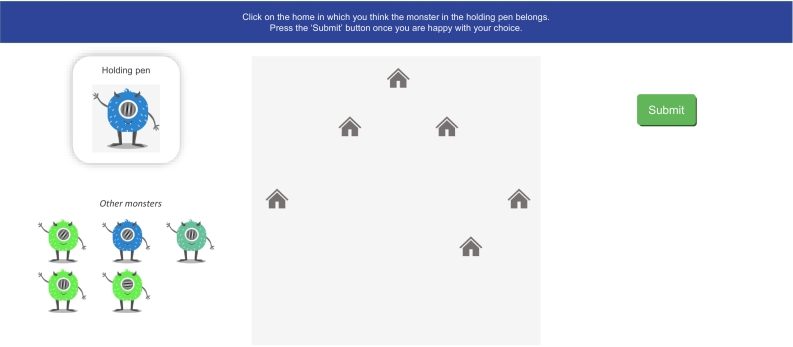
Example of a choice trial display during paired-associate learning. On each choice trial, participants were presented with one monster in the “Holding Pen” on the left of the screen. Participants were instructed to click the house in which they thought the monster lived. They could amend their choice as desired, and all clicks were recorded. Participants were instructed to click “Submit” once they were happy with their choice. The remaining five stimuli were visible under the heading “Other monsters” in the bottom left-hand corner of the screen. Their arrangement was randomised on each page load. Once the response was submitted, participants received feedback on the trial and were shown the monster in its correct home on the map.

The main PAL phase consisted of two trial types: *observational* trials, in which participants were shown each monster's correct home one by one, and *choice* trials, in which participants were presented with one monster and submitted the house in which they thought it lived, providing us with data to analyse. An example choice trial display is shown in Fig. 4.

Observational trials and choice trials were presented in separate blocks, wherein every block contained one trial for each of the 6 stimuli in the set. The order of stimuli was randomised within each block. In total, there were 5 blocks of choice trials, each preceded by two blocks of observational trials. Throughout the PAL task, the neighbourhood map was visible on-screen.In each observational trial, the home whose resident was about to be revealed was cued with a grey border for 1000 ms. The resident monster was then shown in the home for 3000 ms before disappearing. The next home was cued after a 1000 ms break.

After submitting their response on each choice trial, participants received corrective feedback, and were shown the monster in its correct home. Further details on PAL task procedure are provided in Appendix C.

##### 2.1.3.3 Generalisation task

After completing the PAL task, participants completed a single generalisation trial. They were told a new monster had moved to the neighbourhood, and had to choose where it should live on the map. The new monster was shown in the holding pen and there were two new homes to choose from in the locations indicated by red houses in Fig. 3. The monster's colour and eye orientation were both as-yet unseen values.

In the aligned condition, the monster's position within feature space corresponded to the position of one of the presented houses. Details on the generalisation task are provided in Appendix C.

## 3 Results

To evaluate how each condition impacts learning we examine two different measures across PAL blocks: *proportion correct* and mean *distance error*. Proportion correct is the proportion of trials in a block on which a participant mapped the monster correctly. Distance error measures the distance between the chosen home and the correct home. If the monster is placed in the correct home, the response is correct and distance error is 0.

**Fig. 5:**
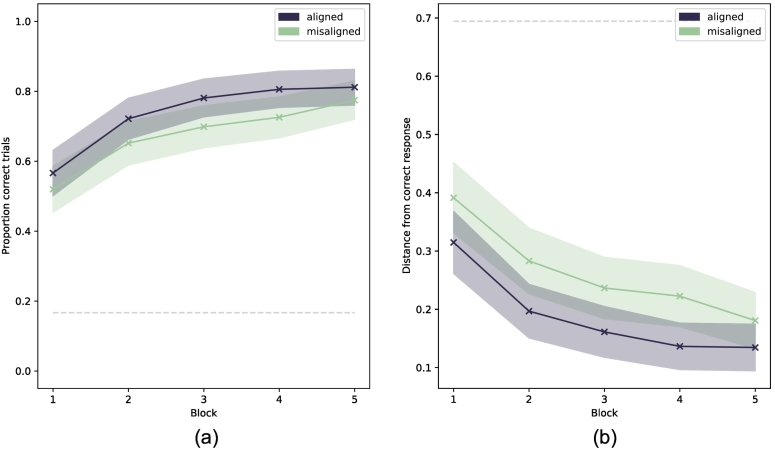
Results by alignment condition for proportion correct and mean distance error by experiment block. Dark blue lines show mean performance for participants in the aligned condition; pale green lines show mean performance for participants in the misaligned condition. Shaded areas show the 95% CI about group means. Dashed lines represent chance performance. (For interpretation of the references to colour in this figure legend, the reader is referred to the web version of this article.)

Analyses revealed significant main effects of alignment condition on both proportion correct and distance error. Results for both measures are shown in Fig. 5. These support our hypotheses that (i) learning is more successful in the PAL task when spaces are alignable than when they are not, and (ii) participants in the aligned condition place the monster in homes with smaller distance error than participants in the misaligned condition. It is worth noting that misaligned participants take 5 blocks of trials to perform at the same standard reached in block 2 by those in the aligned condition— that is more than double the number of trials.

Results for both dependent variables were analysed using mixed-design ANOVAs. In each case, block was included as a within-subjects factor, and alignment and rotation conditions were included as between-subjects factors. Analyses were conducted using the package **ez** in R (Lawrence & Lawrence, 2016).

In the ANOVA model fitted for block-wise mean proportion correct, significant main effects of alignment condition (*F*(1*,* 439) = 7*.*08*, p* = *.*008*, η*_*p*_^2^ = 0*.*016) and block (*F*(3*.*32*,* 1455*.*99) = 134*.*90*, p < .*001*, η*_*p*_^2^ = 0*.*235) were found.

The ANOVA model for block-wise mean distance error also found significant main effects of alignment condition (*F*(1*,* 425) = 15*.*43*, p* =*< .*001*, η*_*p*_^2^ = *.*034) and block (*F*(3*.*30*,* 1450*.*57) = 118*.*59*, p < .*001*, η*_*p*_^2^ = *.*213).

No other terms had significant effects. Full results for both ANOVAs are provided in Appendix E.1.

Our findings in the generalisation trial support the prediction that participants who learn to align across systems are able to generalise to unseen mappings between the alignable structures. 131 of the 222 participants in the aligned condition (59.0%) selected the correct house for the unseen monster.^3^ This result is significantly above chance for *α* = 0*.*05 (*χ*^2^(1) = 7*.*21*, p* = *.*007). No significant difference was found between the rotated and unrotated aligned subconditions (*χ*^2^(1) = 0*.*01*, p* = *.*911).

### 3.1 Model-based analyses

There are a range of cognitive strategies participants may use to learn the mapping, each motivating a model in our analysis. We identify the best-fit model for each participant, and compare the winning model counts within aligned and misaligned learning conditions. This allows us to better understand the distributions of learning strategies used in each condition. The strategy and implementation of each model is briefly summarised below, with further details provided in Appendix F.

• **Classifier** The Classifier model makes no use of the 2D space and simply rote-learns an associated house for each monster. The Classifier is a multilayer perceptron (MLP) that takes as input a monster's feature coordinates and outputs a categorical distribution of the probability of selecting each house.
• **Regression** The Regression model maps monsters into the 2D space of the neighbourhood, demonstrating an appreciation of the continuous nature of the feature spaces. The Regression model is a MLP that takes as input a monster's feature coordinates and outputs the predicted 2D coordinates. The probability of selecting a house is determined by its proximity to the model output; the closer the house, the higher the probability of selection.
• **Regression + Aligner model** The Regression + Aligner model also maps monsters into the neighbourhood, with an added assumption that the systems of houses and monsters should be aligned. This involves a bidirectional mapping between systems, visualised in Fig. 1, and additional loss terms which encourage the alignment of distributions between entire systems once mapped into the same domain. Thus, it updates its internal representations on each trial based on trial feedback, as the Regression model does, and is additionally guided by its efforts to map the structural relationships within entire systems.
• **Random** A Random model was included as a control. The probability of selection was randomly distributed across house options. It did not learn, and no hyperparameters were tuned to the data.

Each model type was fitted for every participant, to see how well it could replicate their behaviour in the PAL task. Models' hyperparameters were selected to minimise the total negative log likelihood (NLL) of the participant's submitted responses across all trials. The sequence of inputs in model training was matched to that seen by the participant during the experiment. This stimulus sequence included choice and observational trials. Observational trials were masked from the NLL calculation in hyperparameter optimisation. The best fitting model for each participant was the one with the lowest AIC model selection statistic, which accounts for both fit and the number of hyperparameters. Details of hyperparameter optimisation and model training are provided in Appendix G.

We find that the majority of participants are best fitted by the Regression.

**Fig. 6:**
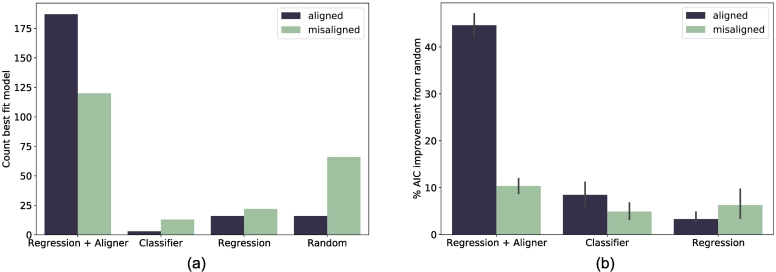
Results of participant-wise model fitting. (a) The count of the number of participants for whom each model type best fitted their trial responses, according to the AIC model selection statistic. (b) The improvement of each participant's best-fitting model AIC on the random model. The majority of participants in both conditions were best fitted by the model which included a system alignment mechanism. AIC performance of the Regression + Aligner model was superior for participants in the aligned condition than those in the misaligned condition, as misaligned participants had to abandon this strategy to learn the task.

+ Aligner model (Fig. 6a), both in aligned (84*.*2%, *χ*^2^(3) = 417*.*46, *p <* *.*001) and misaligned (54*.*3%, *χ*^2^(3) = 130*.*29, *p < .*001) conditions. This supports the hypothesis that participant responses are guided by system alignment, even in the misaligned condition where this strategy is not helpful. However, among participants best fit by the Regression + Aligner model, improvement over the random model for aligned participants was greater than for misaligned participants (Fig. 6b).

## 4 Discussion

The contributions of this study are two-fold: first, the behavioural experiment provides evidence that humans benefit from system alignability when learning to map between spaces, both in terms of the efficiency of learning and the ability to generalise to unseen examples. Secondly, modelling results demonstrate that a system alignment mechanism best accounts for human learning in this task.

The experimental results suggest that aligned spaces facilitate more efficient cross-system learning than misaligned spaces. In the context of Roads and Love (2020)’s finding that spaces derived from unimodal distributional semantics are alignable across modalities, this suggests that system alignment could support cross-modal learning in the real-world. Our significant result for the generalisation task suggests that alignable spaces could facilitate asynchronous integration of multi-modal information in human concept learning (Fourtassi & Dupoux, 2016; Samuelson et al., 2011; Socher et al., 2013). Future work could explore how alignment applies to different domains and types of relations.

The model-fitting results suggest that an offline system alignment mechanism may be recruited in learning associations between systems. Models which performed alignment via an unsupervised loss term were superior on AIC for the majority of participants. In the context of indeterminacy of reference (Quine, 1960) and often infrequent supervised learning episodes (Lieven, 1994), the incremental benefit of an unsupervised alignment loss term suggests a place for alignment in explanations of human concept acquisition. The relative success of the Regression + Aligner model in fitting participant responses in the misaligned condition suggests that learners may default to alignment mechanisms even when systems are not alignable, making errors consistent with this approach. In the context of concept learning, system alignment mechanisms could provide an account of how amodal concept representations incorporate information from different modalities (Patterson, Nestor, & Rogers, 2007; Popham et al., 2021; Ralph, Jefferies, Patterson, & Rogers, 2017).

This study explored the role of alignment signals in supervised learning. Future work may investigate how alignment is used in more ecological multimodal learning contexts, where signals are noisier. Cross-situational learning, for example, provides participants with weak supervision across multiple training episodes (Smith & Yu, 2008; Yu & Smith, 2007), and has been found to be enhanced by semantically themed encoding contexts (Chen & Yu, 2017). Investigating the impact of alignability in a weakly-supervised context would develop our understanding of system alignment's utility the real-world.

The scale of ecological alignment problems is much larger than those tested here, but the possibility remains that established learning processes are supplemented by system alignment. Indeed, larger systems have richer signals for alignment (Goldstone & Rogosky, 2002; Roads & Love, 2020). The relatively small effect size here may be attributed to the task's low difficulty: with only 6 items, the task was intended to be learnable for most participants even in the misaligned case. The benefits of system alignment may increase with problem size, as well as with time to consolidate system mappings in offline replay such as during sleep (cf. Barry & Love, 2021).

In summary, our findings provide evidence for system alignment in accelerating human learning. Together with prior work demonstrating that real-world multimodal spaces are alignable, this opens an avenue to exploring how humans tackle referential ambiguity in concept learning, and how we learn from the statistics of our noisy environments more broadly.

## CRediT authorship contribution statement

**Kaarina Aho:** Methodology, Investigation, Formal analysis, Software, Visualization, Writing – original draft, Writing – review & editing. **Brett D. Roads:** Methodology, Supervision, Visualization, Writing – review & editing. **Bradley C. Love:** Conceptualization, Methodology, Funding acquisition, Supervision, Project administration, Visualization, Writing – original draft, Writing – review & editing.

## Figures and Tables

**Fig. B.7:**
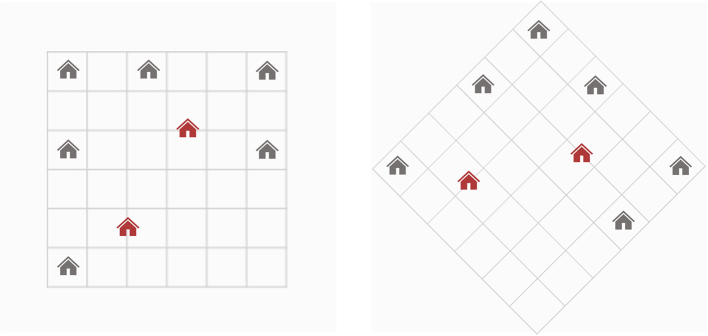
The unrotated (left) and rotated (right) neighbourhood maps. Participants were assigned to a rotation condition at the beginning of the experiment, and learned where each monster lived on their assigned map through paired-associate learning. The map of grey houses was visible to participants throughout the experiment. The red houses were only shown at the end of the experiment, to evaluate zero-shot generalisation based on system alignment. Grid lines are shown for reference only, and were not visible during the experiment. (For interpretation of the references to colour in this figure legend, the reader is referred to the web version of this article.)

**Fig. B.8:**
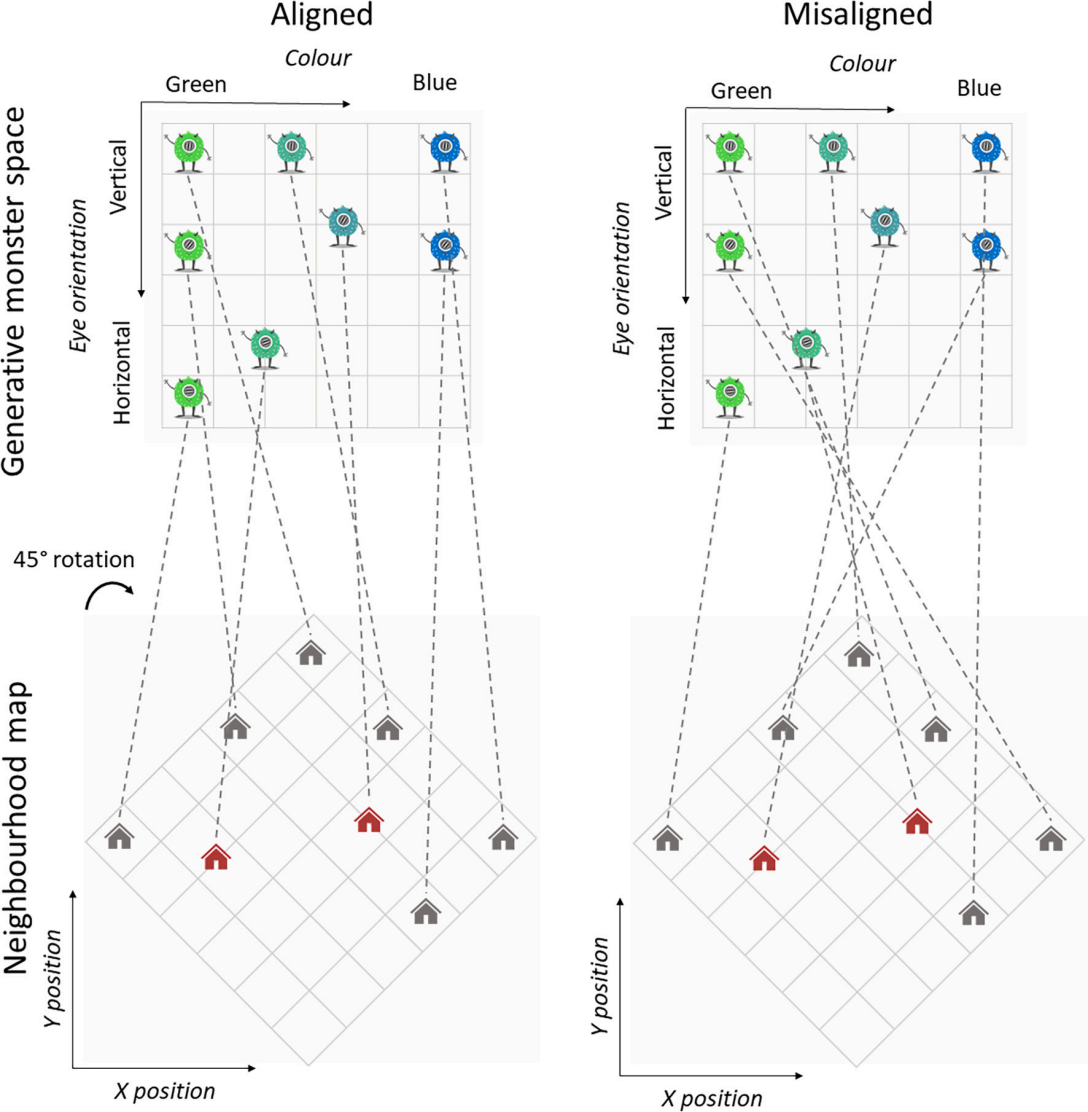
Visualisation of mappings for the aligned and misaligned conditions in the rotated subcondition. In the aligned condition, similarity relationships are still upheld between mapped monster-house pairs; the houses' positions in the neighbourhood map are simply rotated about the map's centre. Monster-house mappings in the misaligned condition remain random.

**Table D.1:** Distribution of participants across conditions pre- and post-application of exclusion criterion. Proportions of each condition in the total pre- and post-criterion samples respectively are shown in parentheses.

		Pre-criterion	Post-criterion
Aligned	Unrotated	123 (0.249)	110 (0.248)
		124 (0.252)	112 (0.251)
Misaligned	Rotated	123 (0.249)	106 (0.239)
		123 (0.249)	115 (0.262)
	Total N	493	443

**Fig. D.9:**
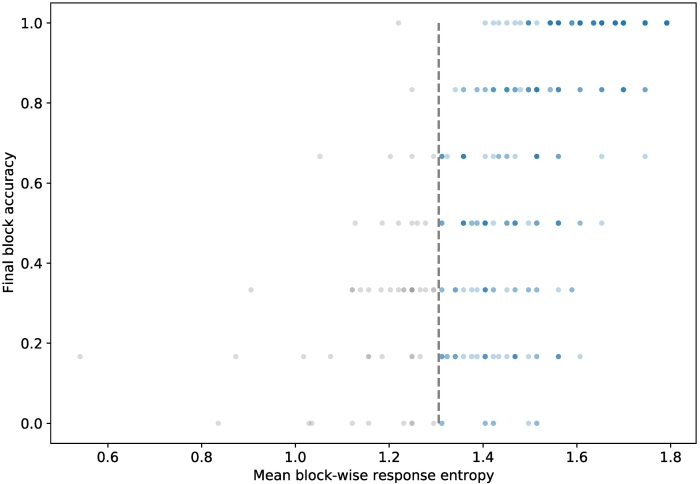
Relationship between final block proportion correct and mean block-wise response entropy for all participants. Grey points represent excluded participants; blue points represent remaining sample after entropy threshold is applied. (For interpretation of the references to colour in this figure legend, the reader is referred to the web version of this article.)

**Table E.2:** Repeated-measures ANOVA for block-wise proportion correct. df = degrees of freedom; *ϵ* = Huynh-Feldt correction factor for df, required by the violation of the sphericity assumption as indicated by Mauchly's test of sphericity, η_*p*_^2^= partial η^2^ effect size.

Predictor	df	*ϵ*	F	p	η_*p*_^2^
Alignment condition	(1*,* 439)		7*.*08	*0.*008 *	*0.*016
Rotation condition	(1*,* 439)		0*.*38	*0.*536	*0.*001
Alignment x Rotation	(1*,* 439)		0*.*19	*0.*661	*< 0.*001
Block	(3*.*32*,* 1455*.*99)	0*.*83	134*.*90	*< 0.*001 *	*0.*235
Alignment x Block	(3*.*32*,* 1455*.*99)	0*.*83	1*.*44	*0.*227	*0.*003
Rotation x Block	(3*.*32*,* 1455*.*99)	0*.*83	1*.*04	*0.*376	*0.*002
Alignment x Rotation x Block	(3*.*32*,* 1455*.*99)	0*.*83	2*.*07	0*.*095	*0.*004

**Table E.3:** Results for repeated-measures ANOVA for block-wise mean distance error. df = degrees of freedom; *ϵ* = Huynh-Feldt correction factor for df as required by the violation of sphericity assumption according to Mauchly's test of sphericity; η_*p*_^2^ = partial *η*^2^ effect size.

Predictor	df	*ϵ*	F	p	η_*p*_^2^
Alignment condition	(1*,* 439)		15*.*43	*< 0.*001 *	*0.*034
Rotation condition	(1*,* 439)		0*.*32	*0.*570	*0.*001
Alignment x Rotation	(1*,* 439)		0*.*06	*0.*805	*< 0.*001
Block	(3*.*30*,* 1450*.*57)	0*.*83	118*.*59	*< 0.*001 *	*0.*213
Alignment x Block	(3*.*30*,* 1450*.*57)	0*.*83	1*.*33	*0.*262	*0.*003
Rotation x Block	(3*.*30*,* 1450*.*57)	0*.*83	0*.*84	*0.*479	*0.*002
Alignment x Rotation x Block	(3*.*29*,* 1450*.*57)	0*.*83	2*.*39	*0.*061	*0.*005

**Table E.4:** Results for block-wise accuracy, all given as percentages.

Block	Aligned (*N* = 222)			Misaligned (*N* = 221)		
	M	SD	95% CI	M	SD	95% CI
1	56*.*6	49*.*6	[50*.*1*,* 63*.*1]	52*.*0	50*.*0	[45*.*3*,* 58*.*6]
2	72*.*1	44*.*8	[66*.*2*,* 78*.*0]	65*.*2	47*.*7	[58*.*9*,* 71*.*4]
3	78*.*1	41*.*4	[72*.*6*,* 83*.*5]	69*.*8	45*.*9	[63*.*8*,* 75*.*9]
4	80*.*6	39*.*6	[75*.*3*,* 85*.*7]	72*.*5	44*.*6	[66*.*7*,* 78*.*4]
5	81*.*6	39*.*1	[76*.*0*,* 86*.*3]	77*.*5	41*.*8	[71*.*9*,* 83*.*0]

**Table E.5:** Results for block-wise mean distance error, all quoted as Euclidean distances on the neighbourhood map grid, scaled such that the maximum possible distance error was $\sqrt{2}$.

Block	Aligned (N = 222)			Misaligned (N = 221)		
	M	SD	95% CI	M	SD	95% CI
1	0*.*315	0*.*407	[0*.*261*,* 0*.*368]	0*.*391	0*.*460	[0*.*331*,* 0*.*452]
2	0*.*197	0*.*351	[0*.*151*,* 0*.*243]	0*.*282	0*.*429	[0*.*226*,* 0*.*340]
3	0*.*161	0*.*333	[0*.*117*,* 0*.*205]	0*.*237	0*.*401	[0*.*183*,* 0*.*290]
4	0*.*136	0*.*304	[0*.*096*,* 0*.*176]	0*.*222	0*.*399	[0*.*170*,* 0*.*275]
5	0*.*134	0*.*305	[0*.*094*,* 0*.*175]	0*.*181	0*.*366	[0*.*132*,* 0*.*229]

**Table E.6:** Results for generalisation performance of participants in aligned and misaligned conditions on the generalisation trial. The “correct” house is the one in which the monster would reside if the monster set were aligned with the house set. Aligned participants perform significantly better than chance in a Chi-square goodness of fit test, as quoted in the main paper body (χ^2^(1) = 7.21, p = .007). Misaligned participants, for whom the correct mapping is not aligned in this way, and who therefore have no way of knowing which house is arbitrarily assigned as the correct one, perform no differently from chance in a Chi-square goodness of fit test, as expected (χ^2^(1) = 0.005, *p* = .946).

	Aligned	Misaligned
Correct	131 (0.590)	111 (0.502)
Incorrect	91 (0.410)	110 (0.498)
Total N	222	221

**Fig. F.10:**
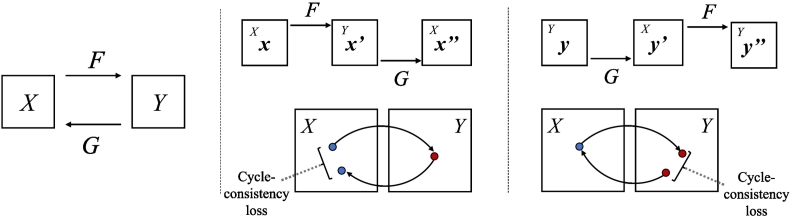
Illustration of cycle consistency loss $L_{\mathrm{cyc}}$, adapted from Zhu, Park, Isola, and Efros (2017). The Aligner model is comprised of two MLPs *F*(*.*) and *G*(*.*), visualised in the leftmost panel. $L_{\mathrm{cyc}}$ measures the average distance between each point in its original space, ***x***, and its reconstruction in the same space ***x***^′′^ generated by the mapping ***x***^′′^ = *F*(*G*(***x***)).

**Fig. F.11:**
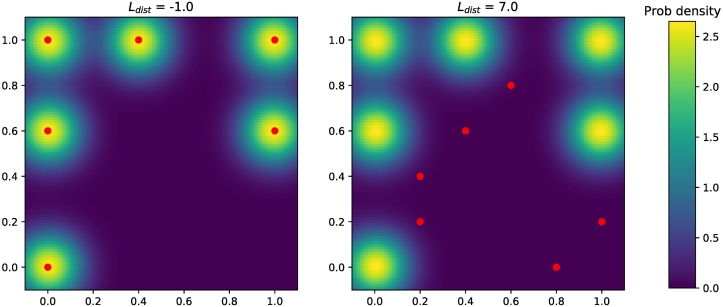
Visualisation of distribution loss $L_{\mathrm{dist}}$ for a low (left) and high (right) loss mapping. Red points represent ***X′***, overlaid on a heatmap representing the probability density of *GMM*_*Y*_. (For interpretation of the references to colour in this figure legend, the reader is referred to the web version of this article.)

## Appendix A Data and code

Data from this study are available at https://osf.io/95md4. Code for data analysis and modelling are available on GitHub at https://github.com/kaarinaaho/learning_alignment.

## Appendix B Stimulus details

### B.1 Neighbourhood stimuli

The rotation condition was included to control for the possibility that participants could align privileged axes instead of whole spaces. The relative positions of houses on the map were kept constant across all participants and conditions. House positions were rotated 45°clockwise about the centre of the map for the rotated condition (see Fig. B.7).

Aligned and misaligned conditions in the rotated subcondition are visualised in Fig. B.8.

### B.2 Monster stimuli

Monster stimuli were generated in the free and open-source graphics editor Krita (version 4.2.2). All monsters had identical body shapes and details

#### B.2.1 Eye orientation

Sinusoidal orientation gratings (or Gabor patches) with a fixed spatial frequency of 5Hz were used as the monsters' eyes. The minimum rotation from horizontal was 5°, and the maximum was 85°. Prior studies have demonstrated that just noticeable differences (JND) in orientation are smaller than 1° (Vogels & Orban, 1985). The minimum difference between Gabor patch angles sampled for our stimuli was 32°for main trial stimuli and 8°for generalisation stimuli.

#### B.2.2 Body colour

This study required that stimuli could be generated at perceptually uniform intervals in the colour dimension, and that the colour values for neighbouring stimuli were perceptually distinct. To meet these criteria, we sampled colours along a linear trajectory in CIECAM02 Uniform Colour Space (CAM02-UCS) (Moroney et al., 2002). CAM02-UCS is a state-of-the-art uniform colour space, which outperforms previous spaces in modelling perceptual distances (Luo, Cui, & Li, 2006). The linear path in CAM02-UCS and corresponding colour scheme were generated using the viscm tool (Van der & Smith, 2015).

For the main trials, we took 6 equally spaced values from this linear trajectory in CAM02-UCS. The CAM02-UCS and its predecessors were designed such that 1 unit distance in the space corresponds to a JND in perception (Mokrzycki & Tatol, 2011). Kuehni (2016) investigated the relationship between JND in colour and the distances in CIECAM02-UCS experimentally, finding that 0.5 units in CAM02-UCS on average corresponded to a JND. Luo et al. (2006) demonstrates colour difference perceptibility in CAM02-UCS by plotting chromatic discrimination ellipses in the space, demonstrating that no difference thresholds perception distances in this space exceed 5 (Luo & Rigg, 1986; Melgosa, Hita, Poza, Alman, & Berns, 1997). The ∆E between our colours in CAM02-UCS, calculated as the Euclidean distance in the space (Luo et al., 2006), is 12.3 - greater than even the most conservative JND values.

## Appendix C Procedure details

### C.1 PAL task

Prior to each set of observational blocks, participants landed on a break screen which prompted them to click a “Continue” button to play the observational blocks.

Once a participant submitted their response for a choice trial, a feedback screen indicated whether their response had been correct or incorrect. If correct, participants advanced to the next trial automatically after 3000ms. If incorrect, participants were prompted to click on the correct home which was highlighted with a grey box. Once they had clicked the correct home, they advanced to the next trial.

### C.2 Generalisation task

The instructions stated that the homes that they had been using in the previous trials would be visible on the map, but were not options for the new monster as they were already occupied. The trial screen was almost identical to the PAL trial screen, but the “Other monsters” grid was removed and the homes that were used for the PAL task were greyed out and unclickable. The monster-house pair was randomly selected from the two possibilities for each participant. Participants clicked on their choice of home for the monster, and submitted their answer. They received no feedback for this trial, and were taken straight to the debriefing page.

## Appendix D Identifying poor engagement

If a participant was making an earnest attempt at the task, we would expect their responses to be distributed near-uniformly across the house options. Participants whose responses were poorly distributed across the space might have repeatedly submitted the same house or alternated between a small number of houses, indicating poor engagement with the task. We sought to exclude poorly engaged participants from the analysis. Our exclusion criterion was based on the average *entropy* of a participant's responses across blocks, ${\bar{H}}_{b}$, which is maximised by a uniform distribution of responses across house options. We excluded the 10% of participants with the lowest ${\bar{H}}_{b}$.

For each participant on each block of trials *b*, we calculated the entropy of the response distribution across options using *H*_*b*_ = $\sum_{i=1}^{6}$P(X_*i*_)log_2_(P(X_*i*_)) where P(X_*i*_) was the probability of the participant selecting house *i* in block *b*, calculated as $P\left(X_{i}\right)=\frac{\sum_{t=1}^{6}\left[X_{t}=i\right]}{6}$ for trial *t* = (1*, ..,* 6) in block *b*. ${\bar{H}}_{b}$ for each participant was the mean of *H*_*b*_ taken across all the experimental blocks. To assess ${\bar{H}}_{b}$as a criterion for participant engagement, we examined the relationship between ${\bar{H}}_{b}$and performance in the final block of trials. If ${\bar{H}}_{b}$were a sensible measure of engagement, we would expect a relationship between low values of ${\bar{H}}_{b}\;$and poor performance on the final block of trials, indicating that participants whose responses were not evenly distributed across the space of house options were not learning the task as well as others. This investigation was performed blindly with respect to experimental condition. The plot in Fig. D.9 demonstrates that there is a strong positive correlation between ${\bar{H}}_{b}$and proportion correct in the final block of responses (*r*_*p*_ = 0*.*748*, p < .*001). Excluding the bottom 10% of participants yielded an exclusion threshold ${\bar{H}}_{b}$ *<* 1*.*31, visualised in Fig. D.9.

The distribution of participants across conditions pre- and post-application of the entropy threshold is shown in Table D.1. A *χ*^2^ test comparing the proportions of participants by condition in the pre- and post-criterion samples reveals no significant difference in the impact of the entropy filter between conditions (*χ*^2^(3) = 0*.*135*, p* = *.*987).

Following the random assignment of participants to conditions, a one- way ANOVA after exclusions are applied finds no significant difference in participant ages between conditions (*F*(3*,* 439) = 0*.*523*, p* = *.*666). A *χ*^2^ test also finds no significant difference in the proportions of females between conditions (*χ*^2^(2) = 0*.*025*, p* = *.*987).

## Appendix F Model details

### F.1 Classifier

The classifier was a multi-layer perceptron (MLP), comprised of an input layer, ReLU activation function, one fully-connected hidden layer of size 100 and output layer of size 6, corresponding to the *n* = 6 homes in which a stimulus could be placed on each trial. The input to the classifier was the 2D coordinate vector of the stimulus in feature space, ***x***, normalised such that *x*_*d*_ ∈ (0, 1) for *d* ∈ {1, 2}. The output vector was fed into a softmax function with temperature parameter *T* to produce a probability distribution across classes.

### F.2 Regression model

The regression model was an MLP, *F*(*.*), comprised of an input layer, ReLU activation function, one fully-connected layer of size 100 and output layer of size 2. The input to the model was the coordinate vector of the stimulus in feature space, ***x***, normalised such that *x*_*d*_ ∈ (0, 1) for *d* ∈ {1, 2}. A sigmoid activation function was applied to model outputs to constrain output values such that *y*_*d*_ ∈ (0, 1) for *d* ∈ {1, 2}. In other words, the MLP performed a mapping *F*: *X* → *Y* from stimulus space *X* to house space *Y*. To generate a probability distribution across house options, the Euclidean distance between the model output and each house option was subtracted from $\sqrt{2}$, (the maximum distance between points in the normalised space), yielding a measure of similarity which took values in range (0*,* $\sqrt{2}$,). If the model had mapped a stimulus perfectly onto a house, this transformation would return its maximum value of $\sqrt{2}$, and conversely if a stimulus was mapped as far as possible from a house the value would be 0. The resultant distributions were fed into a softmax function with temperature parameter *T* to generate a probability distribution across houses for the stimulus according to the model.

### F.3 Regression + aligner model

While the Regression model consisted of one MLP *F*(*.*), which mapped from stimulus domain *X* to neighbourhood *Y*, the Regression + Aligner model consisted of two MLPs: *F*(*.*) and *G*(*.*). These perform mappings *F*: *X* → *Y* and *G*: *Y* → *X* respectively. This is visualised in the leftmost panel of Fig. F.10. *F*(*.*) and *G*(*.*) had the same structure as the MLP *F*(*.*) described above for the Regression model.

The aligner also minimised two additional unsupervised loss components: a *cycle consistency loss* term ($L_{\mathrm{cyc}}$) and a *distribution loss* term ($L_{\mathrm{dist}}$). Inspired by the work of Zhu et al. (2017), $L_{\mathrm{cyc}}$ is defined as the mean Euclidean distance between input stimuli ***X*** and the recovered estimates ***X*** ^′′^, generated by mapping via both MLPs: ***X*** ^′′^ = *G*(*F*(***X*** )). This is visualised in Fig. F.10.

$L_{\mathrm{cyc}}$ included the parallel loss term for the mapping of all ***Y*** to ***Y*** ^′′^. This makes the total cycle consistency loss:

$${\bar{L}}_{\mathrm{cyc}}=\frac{1}{2}(E_{x}[\left\|X-X^{\prime \prime }\right\|+E_{y}\left[\left\|Y-Y^{\prime \prime }\right\|\right])$$

$L_{\mathrm{dist}}$ is visualised in Fig. F.11. In space *Y*, it is defined as the mean negative log likelihood (NLL) of all *F*(***X***) as samples from a Gaussian mixture model comprised of 2D Gaussian kernels placed on ***Y*** (*GMM*_*Y*_ ). $L_{\mathrm{dist}}$ is minimised when all *F*(***X***) are mapped directly onto ***Y***. The Gaussian mixture model is defined as follows:

$${\mathrm{GMM}}_{Y}=\frac{1}{6}\sum_{j=1}^{6}N\left(y;y_{j},\sigma I_{2}\right),$$

where *σ*=0.1. The total distribution loss is the mean of the NLL of ***Y*** ^′′^ as a sample from *GMM*_*Y*_ and the NLL of ***X*** ^′′^ as a sample from *GMM*_*X*_. As both $L_{\mathrm{cyc}}$ and $L_{\mathrm{dist}}$required exposure to the whole space of stimuli, the unsupervised loss terms were not introduced until after the first block of observational trials in model training, where *t >* 6. *λ*_*cyc*_ and *λ*_*dist*_ specified the weights of the cycle consistency and distribution loss terms respectively, relative to the supervised loss term. On each trial in model training, the total loss term was:

$$L=\frac{1}{2}\left({\mathrm{NLL}}_{y_{t}}\left(x_{t}^{'}\right)+{\mathrm{NLL}}_{x_{t}}\left(y_{t}^{'}\right)\right)+\lambda_{\mathrm{cyc}}{\bar{L}}_{\mathrm{cyc}}+\lambda_{\mathrm{dist}}{\bar{L}}_{\mathrm{dis}t}$$

where:

λcyc=λcyc,ift>60,otherwise

λdist=λdist,ift>60,otherwise

## Appendix G Hyperparameter optimisation

All models were built and trained using **pytorch**. Model weights in all cases were initialised with Xavier uniform initialisation. On each trial, models performed 30 update steps using stochastic gradient descent (SGD) with constant *lr*. Multiple steps were required to balance the need for fast learning (owing to the small number of trials) with the instability of high learning rates. Preliminary tests found that 10 gradient steps per trial was the maximum value required for any model to reach optimal performance.

To prevent any probabilities from reaching zero and causing computational issues, we took the maximum of each resultant probability and a small *ϵ* (*ϵ* = 10^−30^), and re-normalised the distribution. In model training, the loss term on each trial was the negative log-likelihood (*NLL*) of the correct response according to this distribution.

Hyperparameter optimisation was performed using the **hyperopt** package in python. Optimisation was performed over 150 evaluations for each model of each participant, using the Tree Parzen Estimator (TPE) method. Preliminary testing found that the success of the Classifier model in learning the task was particularly sensitive to initialisation, while the Regression and Regression + Aligner models were more stable. As such, the classifier was trained three times with each set of hyperparameters tested, and the minimum NLL across the three iterations was taken as the score for those hyperparameters.

In all three models described above, the softmax temperature parameter *T* and learning rate *lr* were hyperparameters. One final hyperparameter, *α*, described each participant's probability of choosing according to the model on any given trial. The probability of choosing a random house was therefore (1 − *α*). Where random variable *Y*_*t*_ is the model's house choice on trial *t*, the probability of a participant choosing house *y* on trial *t* was modelled as:

$$P\left(y\right)=\alpha P\left(Y_{t}=y\right)+\left(1-\alpha \right)\left(\frac{1}{6}\right)$$

The Regression + Aligner model had two additional hyperparameters, *λ*_*cyc*_ and *λ*_*dist*_. This yielded a total number of hyperparameters *k* = 3 for the Classifier and Regression models, and *k* = 5 for the Regression + Aligner model.

## Footnotes

1 Multiple domains can also be contained within a single modality, such as when translating (i.e., mapping) between two natural languages.

2 The mapping of stimulus features onto spatial dimensions (e.g. whether colour or orientation varied in the vertical direction) was randomised by participant. The direction of variation along each spatial axis (e.g. whether a green or blue monster was at the top of the map when colour was mapped onto the vertical) was also randomised independently for each feature dimension.

3 Generalisation analyses are performed on the aligned condition only, as there was no correct response for the misaligned participants. As expected, generalisation results in the misaligned condition were statistically indistinguishable from chance (see Appendix E3).
